# Changes in serum lactate and creatine kinase levels in free-living Geoffroy’s (Phrynops geoffroanus) side-necked turtle captured using funnel traps

**DOI:** 10.1007/s10661-026-15332-y

**Published:** 2026-04-18

**Authors:** Nicole Wirschke de Azevedo, Rafael Martins Valadão, Ana Paula Gomes Lustosa, Patrick Luiz Bola Gonsales, Ana Letícia Rodrigues Marques, Marina Marangoni, Ademar Francisco Fagundes Meznerovvicz, Andriel Gustavo Felichak, Paulo Henrique Braz

**Affiliations:** 1 Department of Veterinary Medicine, Federal University of Fronteira Sul, Realeza, Paraná 85770-000 Brazil; 2 Centro Nacional de Pesquisa E Conservação de Répteis E Anfíbios. Chico Mendes Institute for Biodiversity Conservation, Goiânia, Goiás 74605-090 Brazil; 3 Post-Graduation Program in Health, Welfare and Sustainable Animal Production in the Fronteira Sul, Federal University of the Fronteira Sul, Realeza, Paraná 85770-000 Brazil; 4 Federal University of the Fronteira Sul, Edmundo Gaievski Avenue, 1000, Highway BR 182 - Km 466, Rural Area, PO Box 253, Realeza, Paraná 85770-000 Brazil

**Keywords:** Environmental monitoring, Wildlife, Testudines, Muscle physiology

## Abstract

Capture techniques are widely employed in wildlife monitoring programs, yet confinement and handling may induce transient physiological alterations that complicate interpretation of biochemical parameters. This study evaluated temporal changes in serum lactate and creatine kinase (CK) concentrations in free-living *Phrynops geoffroanus* captured using funnel traps in southern Brazil. Ten adult turtles (5 males, 5 females) were sampled at two time points: immediately after transport from capture sites (T0) and 24 h later (T24). Paired analyses indicated a significant decrease in lactate concentrations from T0 (90.6 ± 44.0 mg/dL) to T24 (38.2 ± 20.8 mg/dL; p = 0.0044), consistent with recovery from acute anaerobic activation. In contrast, CK activity increased significantly from T0 (2,227.8 ± 2,776.9 U/L) to T24 (8,131.0 ± 5,306.7 U/L; p = 0.0012), suggesting delayed enzymatic release associated with muscular strain. These divergent temporal patterns reflect distinct physiological mechanisms and biomarker kinetics following capture. The results highlight the importance of considering sampling latency when interpreting post-capture biochemical values in ecological studies. Funnel traps proved operationally effective, but confinement duration should be carefully managed to minimize physiological stress. Incorporating temporal context into capture protocols can improve the accuracy of physiological assessments in environmental monitoring programs.

## Introduction

Wildlife management and ecological monitoring often require capture and restraint procedures to assess species composition, distribution, and health status within natural environments (Silveira et al., 2010; Roque et al., 2016). Although such procedures are essential for conservation and monitoring efforts, capture-related stress may induce transient physiological changes in free-ranging animals.

In reptiles, capture and restraint elicit acute compensatory physiological mechanisms associated with escape attempts and intermittent hypoxia (e.g., breath-holding), which favor a transient shift toward anaerobic metabolic pathways (Harms et al., 2003; Snoddy et al., 2009). These responses can increase circulating lactate concentrations and may contribute to subsequent elevations in muscle-associated enzymes such as creatine kinase (CK), reflecting exertion-related muscle membrane disruption (Hellmuth et al., 2012).

CK is an enzyme with high activity in skeletal and cardiac muscle tissues and is considered a specific biomarker of myocellular damage in reptiles, as in mammals. Muscle fiber microlesions promote the release of CK into circulation, typically with a delayed peak relative to the inciting event, owing to its longer biological half-life and slower clearance (Allison, 2024; Campbell, 2024). In contrast, lactate is produced rapidly during anaerobic glycolysis when oxygen availability is limited. Under intense exertion or forced submersion, reptiles rely on intramuscular glycogen as the primary substrate, producing lactate as a metabolic by-product that initially accumulates in muscle tissue before entering the bloodstream (Gleeson, 1991; Harms et al., 2003; Snoddy et al., 2009).

This study evaluated freshwater chelonians of the species *Phrynops geoffroanus* (order Testudines, family Chelidae), which are widely distributed across rivers and streams throughout South America. Commonly known as the “Geoffroy’s side-necked turtle,” *P. geoffroanus* exhibits reddish coloration with black markings on the ventral cervical region and along the lateral portion of the trunk during the juvenile stage; these colors subsequently fade in adulthood (Bujes, 2010; Rhodin et al., 2021).

Among the available capture techniques for freshwater turtles, funnel traps are frequently recommended due to their relatively high survival rates compared to net-based systems. However, the physiological consequences of confinement within these traps, particularly regarding muscular exertion and metabolic stress, remain poorly quantified. Therefore, this study aimed to evaluate serum lactate and CK concentrations at two time points following capture using funnel traps, contributing to the refinement of capture protocols in environmental monitoring programs.

## Materials and methods

### Study area and capture procedures

A total of 10 free-living *Phrynops geoffroanus* turtles were captured using funnel traps installed along the margins of the Silva Jardim, Floriano, and Iguaçu Rivers. Traps measured 80 × 46 × 20 cm, had a 15 mm mesh, and were baited with a mixture of ground beef, bovine liver, canned sardines, pineapple, and dry cat food. The baits were positioned in such a way that prevented the turtles from accessing and consuming the contents. To avoid drowning, the traps were fitted with a flotation system that allowed approximately 30% of their structure to remain above the water surface.

### Transport and blood sampling

After capture, the animals were manually restrained, identified, and transported to the research base of the Brazilian Institute for Biodiversity Conservation. Transport time from trap retrieval to arrival at the field base ranged from approximately 30–60 min. During transit, animals were maintained undisturbed in shaded containers. Because lactate can change over short time scales with activity and recovery, this interval was considered in the interpretation of T0 values.

Blood was collected from the dorsal cervical venous sinus within the limits of 0.5 to a maximum of 0.8% of body weight. The puncture site was disinfected with povidone–iodine solution, and a 3 mL syringe fitted with a 21G × 1¼ inch needle (30 × 0.8 mm) was used. Needle gauge was selected to balance adequate flow and minimize hemolysis/lymph contamination in chelonians, following published venipuncture guidance. All samples were obtained by an experienced operator, avoiding repeated punctures.

### Biochemical analyses

Immediately after collection, whole blood was centrifuged at 5,000 rpm for 5 min. Serum was then aliquoted into sterile microtubes and stored at 4 °C until biochemical analysis. Both lactate and CK were measured using commercial diagnostic kits on an automated biochemical analyzer (Wiener Lab CM 250®) at the Clinical Analysis Laboratory of the Federal University of Fronteira Sul, following the manufacturers’ assay conditions.

### Statistical analysis

All statistical analyses were performed in RStudio (R Core Team 2023, version 4.5.1) using the packages tidyverse, rstatix, lme4, lmerTest, ggpubr, and effsize. Data normality was evaluated with the Shapiro–Wilk test. Given the different kinetics of lactate (rapid change with exertion/recovery) and CK (often delayed response to muscle stress), we did not model direct associations between biomarkers across time points. Comparisons between the two time points were conducted using paired t-tests. Analyses focused on within-individual changes between T0 and T24 for each parameter.

## Results

Shapiro–Wilk tests applied to the paired differences (Δ = T24 − T0) indicated no significant deviation from normality for either creatine kinase (CK) (W = 0.899, p = 0.214) or lactate (W = 0.933, p = 0.478). Therefore, paired t-tests were used to compare biochemical parameters between sampling times. Descriptive statistics for all parameters are presented in Table 1.

**Table 1:** Descriptive statistics (mean ± SD, minimum and maximum values) for serum creatine kinase (CK) and lactate concentrations at T0 and T24

	Mean ± SD	Minimum	Maximum
CK (U/L)			
T0	2,227.8 ± 2,776.9	187.0	9,108.0
T24	8,131.0 ± 5,306.7	1,375.0	15,025.0
Lactate (mg/dL)			
T0	90.6 ± 44.0	14.7	161.0
T24	38.2 ± 20.8	10.8	70.2

**Fig. 1:**
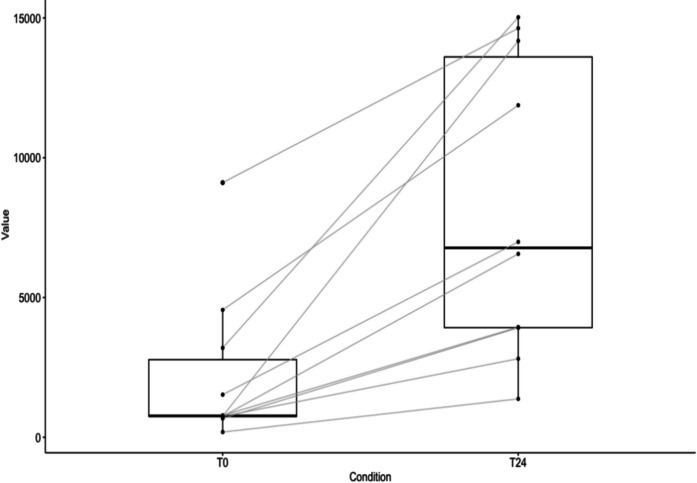
Variation in serum creatine kinase (U/L) levels between T0 and T24 in captured individuals

Serum CK concentrations increased from T0 (2,227.8 ± 2,776.9 U/L; range: 187.0–9,108.0 U/L) to T24 (8,131.0 ± 5,306.7 U/L; range: 1,375.0–15,025.0 U/L) (Fig. 1). This increase was statistically supported (paired t-test; df = 9, t = 4.639, p = 0.0012), with a mean difference of 5,903.2 U/L (95% CI: 3,024.7–8,781.7). The magnitude of change was large (Cohen’s d = 1.20; 95% CI: 0.49–1.91).

**Fig. 2:**
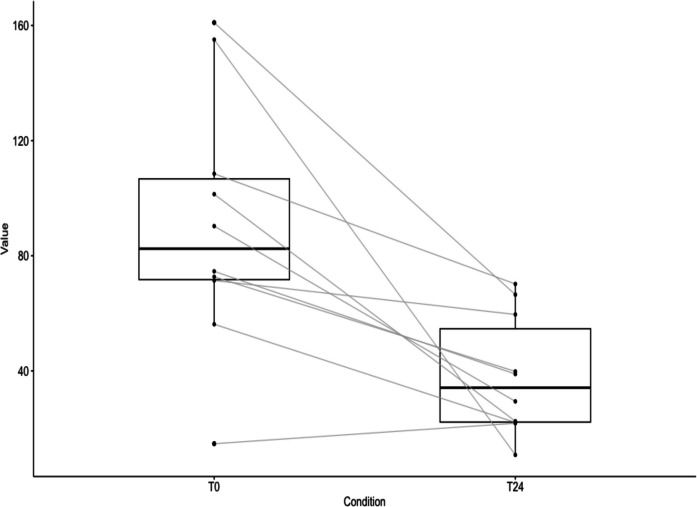
Variation in serum lactate (mg/dL) levels between T0 and T24 in captured individuals

Conversely, lactate concentrations decreased between sampling times, from 90.6 ± 44.0 mg/dL at T0 (range: 14.7–161.0 mg/dL) to 38.2 ± 20.8 mg/dL at T24 (range: 10.8–70.2 mg/dL) (Fig. 2). This reduction was statistically supported (paired t-test; df = 9, t = − 3.774, p = 0.0044), with a mean difference of − 52.4 mg/dL (95% CI: − 83.9 to − 21.0). The effect size was also large (Cohen’s d = − 1.47; 95% CI: − 2.65 to − 0.29).

Descriptive analysis by sex indicated that mean CK values at T0 were higher in males (2,687.8 U/L) than in females (2,155.8 U/L). At T24, females presented higher mean CK concentrations (10,176.6 U/L) compared to males (5,966.5 U/L). For lactate, mean concentrations at T0 were similar between males (81.8 mg/dL) and females (84.7 mg/dL). At T24, lactate decreased in both sexes.

## Discussion

The present study demonstrates distinct temporal patterns for lactate and creatine kinase (CK) following capture of *Phrynops geoffroanus* using funnel traps. While lactate concentrations decreased significantly between T0 and T24, CK values increased over the same interval, suggesting that these biomarkers reflect different physiological processes and operate on different temporal scales.

Lactate is widely recognized as a sensitive indicator of anaerobic metabolism in reptiles (Bennett & Licht, 1972). During intense locomotor activity, escape attempts, or prolonged breath-holding, behaviors commonly observed during capture and confinement, reptiles rely heavily on intramuscular glycogen stores as substrates for anaerobic glycolysis (Gleeson, 1991). This metabolic shift results in rapid lactate production, which may initially accumulate in muscle tissue before diffusing into circulation (Bennett & Licht, 1972).

Experimental studies in reptiles have demonstrated that lactate concentrations can rise within minutes of forced activity and begin declining once aerobic metabolism resumes (Cushman et al., 1976; Harms et al., 2003). In sea turtles, elevated lactate levels have been consistently associated with capture stress and entanglement events, reinforcing its role as a marker of acute exertion (Snoddy et al., 2009).

In the present study, lactate concentrations were elevated at T0 and significantly reduced after 24 h, consistent with metabolic recovery following removal from trap confinement and subsequent rest. Although the precise duration of active exertion inside traps could not be quantified, the initial concentrations are compatible with anaerobic activation during confinement. The decline observed at T24 suggests restoration of aerobic metabolism and physiological re-equilibration. Similar post-capture lactate declines have been documented in marine and freshwater chelonians after cessation of stressors (Harms et al., 2003; Snoddy et al., 2009). Importantly, lactate clearance in reptiles involves both oxidation within skeletal muscle and gluconeogenic pathways, with the relative contribution of each mechanism influenced by temperature, acid–base balance, and species-specific metabolic characteristics (Gleeson, 1991).

In contrast to lactate, CK activity reflects structural compromise of muscle cell membranes and subsequent leakage of intracellular enzymes into the bloodstream (Arguedas, 2024; Vanholder et al., 2000). Unlike lactate, which responds rapidly to changes in metabolic demand, CK typically demonstrates delayed elevation following muscular strain, with peak concentrations often occurring hours to days after the inciting event (Câmara et al., 2020; Hanley et al., 2005; Innis et al., 2014).

In reptiles, CK is considered a useful but context-dependent biomarker of myocellular stress, as values can vary considerably among individuals and are influenced by restraint intensity, handling duration, and capture technique (Allison, 2024; Arguedas, 2024; Campbell, 2024). Experimental restraint studies in sea turtles have demonstrated progressive increases in muscle-associated enzymes over time, even after metabolic indicators such as lactate begin to normalize (Harms et al., 2003).

The consistent increase in CK observed at T24 in this study is therefore physiologically coherent. Rather than representing ongoing metabolic exertion, the delayed elevation likely reflects muscle fiber microdamage sustained during trap confinement and capture handling. This interpretation aligns with the known temporal dissociation between rapid metabolic markers and slower enzymatic responses to tissue stress. The absence of intermediate sampling points precludes determination of the precise timing of peak CK activity; however, the magnitude of change and its consistency across individuals suggest a systemic rather than incidental response.

The divergent trajectories of lactate and CK underscore the importance of considering biomarker kinetics when interpreting post-capture physiological data. Lactate primarily reflects immediate metabolic demand and resolves relatively quickly following cessation of activity, whereas CK may continue to rise after the initial stressor due to delayed enzyme release and clearance dynamics. The absence of direct association between these markers (as expected given their differing biological half-lives and mechanisms) should therefore not be interpreted as physiological independence but rather as temporal dissociation.

Venipuncture-related muscle trauma must also be acknowledged as a potential source of CK variability. In chelonians, blood collection from the dorsal cervical venous sinus requires penetration through relatively thick integument and underlying musculature. Although sampling was performed by an experienced operator without repeated punctures or visible tissue injury, minor localized muscle disruption cannot be entirely excluded. Nevertheless, if venipuncture were the primary determinant of CK elevation, increases would be expected to occur closer to the time of sampling rather than showing a consistent delayed rise after 24 h. Thus, while procedural influence cannot be ruled out, the temporal pattern observed is more consistent with capture-related muscular exertion.

Sex-related descriptive differences were observed in CK values. Baseline CK was higher in males, whereas females exhibited higher mean CK at T24. Similar sex-based variation in muscle-associated enzymes has been reported in chelonians and may be influenced by differences in body composition, activity patterns, or reproductive physiology (Mumm et al., 2019; Yang et al., 2014; Yu et al., 2013). However, given the modest sample size (n = 5 per sex), these patterns should be interpreted cautiously and not as evidence of definitive sex-related physiological differences.

## Conclusions

From an environmental monitoring perspective, these findings highlight the importance of considering sampling latency relative to capture when interpreting biochemical parameters in wild reptiles. Lactate reflects acute metabolic activation and resolves relatively rapidly, whereas CK may continue to increase after the initial stressor due to delayed enzymatic kinetics. Incorporating temporal context into sampling protocols is therefore essential for accurate physiological assessment in ecological studies.

Although the absence of intermediate sampling points limits precise characterization of peak timing and clearance dynamics, the observed temporal dissociation between lactate and CK underscores the need for careful interpretation of post-capture biomarkers. Additionally, the modest sample size (n = 10) suggests that further studies with larger populations and finer temporal resolution are warranted.

Overall, funnel trap confinement in *P. geoffroanus* induced measurable, time-dependent physiological alterations characterized by acute anaerobic activation followed by delayed muscular enzyme elevation. The funnel trap proved operationally effective and required minimal direct handling; however, confinement duration should be carefully managed to minimize physiological stress and ensure animal welfare during ecological monitoring activities.

## Data Availability

All data generated or analysed during this study are included in this published article. The data that support the findings of this study are available from the corresponding author, upon reasonable request.

## Declarations

All authors have read, understood, and have complied as applicable with the statement on “Ethical responsibilities of Authors” as found in the Instructions for Authors.
